# Linear ubiquitination in immunity

**DOI:** 10.1111/imr.12309

**Published:** 2015-06-18

**Authors:** Yutaka Shimizu, Lucia Taraborrelli, Henning Walczak

**Affiliations:** ^1^ Centre for Cell Death, Cancer, and Inflammation (CCCI)UCL Cancer InstituteUniversity College LondonLondonUK

**Keywords:** linear ubiquitination, deubiquitinases, signaling pathways, cell death, inflammation

## Abstract

Linear ubiquitination is a post‐translational protein modification recently discovered to be crucial for innate and adaptive immune signaling. The function of linear ubiquitin chains is regulated at multiple levels: generation, recognition, and removal. These chains are generated by the linear ubiquitin chain assembly complex (LUBAC), the only known ubiquitin E3 capable of forming the linear ubiquitin linkage *de novo*. LUBAC is not only relevant for activation of nuclear factor‐κB (NF‐κB) and mitogen‐activated protein kinases (MAPKs) in various signaling pathways, but importantly, it also regulates cell death downstream of immune receptors capable of inducing this response. Recognition of the linear ubiquitin linkage is specifically mediated by certain ubiquitin receptors, which is crucial for translation into the intended signaling outputs. LUBAC deficiency results in attenuated gene activation and increased cell death, causing pathologic conditions in both, mice, and humans. Removal of ubiquitin chains is mediated by deubiquitinases (DUBs). Two of them, OTULIN and CYLD, are constitutively associated with LUBAC. Here, we review the current knowledge on linear ubiquitination in immune signaling pathways and the biochemical mechanisms as to how linear polyubiquitin exerts its functions distinctly from those of other ubiquitin linkage types.

This article is part of a series of reviews covering Ubiquitination in the Immune System appearing in Volume 266 of *Immunological Reviews*

## Introduction

Ubiquitination (also known as ubiquitylation or ubiquitinylation) is a post‐translational protein modification that consists in the attachment of one or more ubiquitins to a target protein. Ubiquitin is an evolutionarily highly conserved small protein of 76 amino acids (8.6 kDa) 1. Ubiquitination is carried out by three different classes of enzymes: ubiquitin‐activating enzymes (E1s), ubiquitin‐conjugating enzymes (E2s), and ubiquitin‐ligating enzymes (E3s) 2. E1 catalyzes the activation of ubiquitin in an ATP‐dependent reaction to generate a thioester bond between the C‐terminal carboxyl group of ubiquitin and the catalytically active cysteine (Cys) of the E1 enzyme. Subsequently, the activated ubiquitin is transferred to the cysteine residue in the active site of the E2 before the E3 mediates attachment of ubiquitin to the target protein. The human genome encodes only two characterized E1s 3, nearly 40 E2s 4, and more than 600 E3s 5 in the ubiquitin system. According to the presence of particular domains in them, E3s are divided into three groups: homologous to E6‐AP C‐terminus (HECT), really interesting new gene (RING), and U‐box E3s. In the case of HECT E3s, ubiquitin is first transferred to the active cysteine of the E3 and subsequently the E3 adds it to the substrates. RING and U‐box E3s instead directly mediate transfer of ubiquitin from E2 to substrates 2.

Ubiquitin itself has seven lysine (Lys) residues that can function as a linker to another ubiquitin (Lys6, Lys11, Lys27, Lys29, Lys33, Lys48, and Lys63) 1 and normally ubiquitination results in the formation of an isopeptide bond between the carboxyl group of the C‐terminal glycine of ubiquitin and the ε‐amino group of lysine residue of target proteins. Yet, Kirisako *et al*. 6 discovered that ubiquitin can also be conjugated to the amino‐terminal methionine (Met1) of ubiquitin itself. Therefore, there are eight different di‐ubiquitin linkages depending on which residue in ubiquitin is linked to another ubiquitin moiety. Each type of di‐ubiquitin linkages forms a unique three‐dimensional structure. For instance, Lys6‐, Lys11‐, and Lys48‐linked di‐ubiquitins take ‘compact’ structures with intramolecular interfaces. In contrast, Lys63‐ and Met1‐linked di‐ubiquitins adopt an ‘open’ structure 1. The divergent topologies of the different linkages result in distinct biological outcomes as they determine which ubiquitin receptors are recruited to which linkage type and, hence, to which signaling complex. For this reason it is important to determine the type of linkages present in different signaling complexes—or on individual components of such complexes.

So far, the best characterized function of ubiquitin is its degradative role for Lys48‐linked polyubiquitin. E3 ligases attach Lys48‐linked polyubiquitin to a substrate as a degradation label and ubiquitinated substrates are subsequently subjected to degradation by the proteasome 2. Nevertheless, whereas Lys48‐linked polyubiquitin serves as a signal for destruction, the other linkage types play non‐degradative roles. Notably, Lys63‐ and Met1‐linked (i.e. linear) polyubiquitins are crucial for the regulation of diverse signaling pathways 1, 7. When a ubiquitin chain contains multiple types of inter‐ubiquitin linkages, it is called heterotypic or hybrid. This diversity of ubiquitin chains confers multiple functions which emanates from a substrate. Ubiquitination is hence a complex post‐translational modification and offers the possibility to fine‐tune the regulation of biological processes.

Over the past decade, Met1‐linked ubiquitination has been shown to be engaged in a wide range of immune signaling pathways. In this review, we first explain the molecular machinery of its production and its involvement in multiple immune receptor signaling pathways. Subsequently, we focus on its regulation by specific deubiquitinases (DUBs) and ubiquitin binding proteins to highlight the mechanism by which linear ubiquitin is specifically targeted and recognized in cells. Finally, we propose how the complexity of ubiquitination, particularly the probing of linear ubiquitin on target proteins, may be untangled.

## LUBAC

The linear ubiquitin chain assembly complex (LUBAC) is the only enzyme complex identified so far that can generate the linear di‐ubiquitin linkage *de novo* under native conditions 8, 9, 10. LUBAC consists of two RING‐between‐RING (RBR) E3s, which are heme‐oxidized iron‐responsive element‐binding protein 2 (IRP2) ubiquitin ligase‐1 (HOIL‐1) and HOIL‐1‐interacting protein (HOIP), and a third component which is the SH3 and multiple ankyrin repeat domains protein (SHANK)‐associated RBCK1 homology (RH)‐domain‐interacting protein (SHARPIN) 11, 12, 13. Although both HOIL‐1 and HOIP bear an RBR domain, HOIP is the catalytically active subunit of LUBAC, since LUBAC containing inactive HOIL‐1 can still produce linear ubiquitin chains 6. However, HOIP alone is not sufficient to create linear ubiquitin chains as it requires at least one of the other LUBAC components, HOIL‐1 or SHARPIN, to do so 11, 12, 13. This implies that HOIP is self‐inhibitory and that binding to HOIL‐1 or SHARPIN liberates HOIP from auto‐inhibition so that it can generate linear ubiquitin chains. HOIP interacts with the ubiquitin‐like domain (UBL) domain of HOIL‐1 via its ubiquitin‐associated (UBA) domain 6. HOIP has been shown to bind to the UBL domain of SHARPIN via its UBA domain 13. Our group and others proposed that the nuclear protein localization 4‐zinc‐finger (NZF) 2 domain of HOIP contributes to the binding to SHARPIN 11, 12, although it appears to be dispensable according to Tokunaga *et al*. 13. HOIL‐1 and SHARPIN stabilize HOIP as the protein level of HOIP is reduced when either of the components are depleted.

The C‐terminus of HOIP harbors an RBR domain and a linear ubiquitin chain‐determining domain. Together, they represent the minimal catalytic core capable of forming Met1‐linked di‐ubiquitin (Met1‐di‐Ub) 14. Like other members of the RBR E3 protein family, such as Parkin and human homolog of *Drosophila* Ariadne (HHARI), HOIP works as a RING/HECT hybrid E3 15. HOIP first binds to ubiquitin‐conjugated E2 via its RING1 domain. Subsequently, ubiquitin is transferred to a catalytic center in the RING2 domain. The distal ubiquitin moiety binds to the zinc‐finger (ZF) motif in the RING2 domain of HOIP and this binding orients the Met1 residue in the distal ubiquitin toward the catalytic center. In the proximity of the catalytic Cys885, histidine (His) 877 acts as the basic residue to activate the α‐amino group of Met1 as a nucleophile. Activated Met1 attacks the thioester bond which is preformed between the C‐terminus of the proximal Ub and the catalytic cysteine to form the Met1‐di‐Ub linkage 14. Therefore, the specificity for Met1‐di‐Ub formation by HOIP is coordinated between its catalytic core and its interaction with the two ubiquitin moieties to be linked.

## LUBAC in TNF signaling

**Figure 1:**
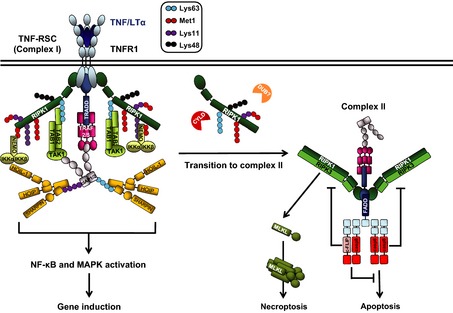
**TNFR** **1 signaling complexes and outputs.** Upon TNF/LTα ligation, TNFR1 trimerizes and recruits TNF‐RSC (complex I) components to activate nuclear factor‐κB (NF‐κB) and MAPK signaling pathways. Different types of ubiquitination (indicated in a graphic legend) coordinate the stability of complex I. Linear ubiquitin chain assembly complex (LUBAC) is recruited to complex I to ubiquitinate RIPK1 and NF‐κB essential modulator (NEMO). Some components of complex I can dissociate and form a secondary complex, complex II. Deubiquitination of Lys63‐linked polyubiquitin on RIPK1 by cylindromatosis (CYLD) favors the transition. Other deubiquitinases (DUBs) can possibly contribute to the transition in a similar manner. Formation of complex II can result in different outcomes. First, when gene activation properly occurs, cFLIP can bind to caspase‐8 (casp8) and block cell death. Second, when gene activation is impaired, caspase‐8 forms a heterodimer and activates itself to elicit apoptosis. By contrast, when caspase‐8 activity is inhibited or when caspase‐8 or FADD is absent, RIPK1 and RIPK3 are activated and RIPK3 phosphorylates mixed lineage kinase domain‐like protein (MLKL), resulting in necroptosis.

The prototypic signaling complex at which ubiquitination is studied is the TNFR1 signaling complex (TNF‐RSC). Unsurprisingly, it was therefore also the study of this complex that led to the discovery that LUBAC forms part of signaling complexes and that linear ubiquitination is crucial for enabling the physiological signaling output of TNFR1 11, 16. Binding of TNF or lymphotoxin‐α (LTα) to TNFR1 results in receptor trimerization and formation of the TNF‐RSC. Upon trimerization of TNFR1, TNF receptor‐associated death domain (TRADD) and receptor‐interacting protein kinase 1 (RIPK1) are independently recruited to the intracellular death domain (DD) of TNFR1 via their respective DDs. TRADD then serves as a platform for recruitment of TNF receptor‐associated factor 2/5 (TRAF2/5), which in turn recruits the two E3s cellular inhibitor of apoptosis 1 and 2 (cIAP1/2). Together, cIAP1 and 2 attach Lys63‐, Lys11‐, and Lys48‐linked polyubiquitin to RIPK1 and to themselves 17, 18, 19. This enables recruitment of LUBAC and a complex consisting of TAK1‐binding protein 1 and 2/3 (TAB1‐TAB2/3)‐transforming growth factor β‐activated kinase 1 (TAK1), referred to as the TAB/TAK complex hereafter. The recruitment of these two complexes in turn allows for the subsequent recruitment and activation of inhibitor of κB (IκB) kinaseα (IKKα) and IKKβ through the NF κB essential modulator (NEMO) with which they form a complex 16, hereafter referred to as IKK complex. This results in activation of canonical NF‐κB and the mitogen‐activated protein kinases (MAPKs) as the gene‐activatory signaling outputs of the TNF‐RSC, leading to upregulation of a plethora of pro‐survival and inflammatory genes. Recruitment of LUBAC to the TNF‐RSC depends on the presence of TRADD and TRAF2 as well as on presence and E3 ligase activity of cIAPs 16. Once LUBAC is recruited to the TNF‐RSC, it attaches linear ubiquitin chains to RIPK1 and NEMO, thereby stabilizing the TNF‐RSC and supporting activation of NF‐κB and MAPKs as in the absence of LUBAC components cells display reduced TNF‐induced NF‐κB and MAPK activation 16 (*Fig.* 1).

Under certain circumstances, TNF can also induce cell death. When the TNF‐RSC, also termed complex I of TNFR1 signaling, is destabilized, a secondary cytoplasmic complex, known as complex II, is formed 20. TNF‐induced cell death signaling emanates from complex II. Deubiquitination of RIPK1 by the DUB cylindromatosis (CYLD) appears to be a prerequisite for the formation of complex II, because ubiquitinated RIPK1 protects cells from TNF‐induced death 21, 22, 23. The biochemistry of the transition from complex I to complex II is at present not fully understood. What we know is that RIPK1, which dissociates from complex I, forms the core of complex II and that it recruits the Fas‐associated protein with DD (FADD) via its own DD. RIPK1‐associated FADD in turn recruits caspase‐8 via its death effector domain, resulting in activation of this apical caspase in the apoptosis signaling pathway. Once activated, caspase‐8 cleaves and activates the BH3‐only protein BID and the downstream effectors caspase‐3 and ‐7 which, together, results in apoptotic death of most cells 8, 24.

When caspase‐8 activity is inhibited at complex II, or when caspase‐8 or FADD are absent from it, another type of cell death can be induced from complex II. This form of cell death is referred to as programmed necrosis or necroptosis and requires the concerted action of two kinases, RIPK1 and RIPK3, together with that of a pseudokinase, mixed lineage kinase domain‐like protein (MLKL). RIPK1 and RIPK3 can be kept from inducing necroptosis when cleaved by caspase‐8. Interestingly, their cleavage by the caspase‐8/c‐FLIP heterodimer, which does not induce apoptosis, appears to be required to block necroptosis 25, 26. Once caspase‐8 activity is impaired, the kinase activity of RIPK1 is responsible for phosphorylation of RIPK3. RIPK3 subsequently phosphorylates MLKL 27. The phosphorylation of MLKL alters its conformation to form an oligomer. MLKL oligomers have been reported to bind to phosphatidylinositol lipids on the plasma membrane and to insert into the membrane to form a pore, resulting in leakage of cellular content and necrotic death 28, 29. Thus, depending on its constitution and the activities of its components, two different signals emanate from complex II and, depending on which of them prevails, either apoptotic or necroptotic cell death ensues.

Importantly, absence of LUBAC components renders cells more sensitive to TNF‐ and LTα‐induced apoptosis and necroptosis 11, 30. It is not entirely clear how LUBAC prevents TNFR1‐mediated cell death. In LUBAC‐deficient cells TNF‐induced cell death indeed coincides with reduced activation of TNF‐induced NF‐κB and MAPKs. However, LUBAC deficiency leads to robust formation of complex II which, intriguingly, is independent of its effects on gene activation 30. Thus, it appears that linear ubiquitination does not maintain resistance to TNF‐induced cell death indirectly via gene activation. Instead, it does so directly by preventing complex II formation through linear ubiquitination in complex I. The specific linear ubiquitination event(s) in complex I, which prevent(s) formation of complex II remain(s), however, unresolved. Independently of the answer to this question, LUBAC and its activity are crucial for maintaining the balance between the gene‐activatory and cell death‐inducing outputs of TNF signaling.

## LUBAC in innate immune signaling

The innate immune system recognizes pathogens via pattern recognition receptors (PRRs). Microbial products, called pathogen‐associated molecular patterns (PAMPs) and danger signals released by damaged host cells, referred to as damage‐ (or danger‐) associated molecular patterns (DAMPs), are recognized by PRRs from immune and non‐immune cells. There are different classes of PRRs with Toll‐like receptors (TLRs) and C‐type lectin receptors being located in the plasma membrane or on endosomes, whereas other PRRs such as retinoic acid‐inducible gene‐I (RIG‐I)‐like receptors (RLR), the absent in melanoma 2 (AIM2)‐like receptors (ALRs) and the nucleotide‐binding domain and leucine‐rich repeat containing (NLR) proteins, are located in the cytosol. Activation of PRRs by PAMPs and DAMPs triggers production of pro‐inflammatory factors responsible for recruitment and activation of immune cells 31, 32. In this way, the PRRs elicit a first line of defense against pathogens.

### TLR/IL‐1R signaling

TLRs are receptors that recognize a variety of pathogens, such as bacteria, fungi, viruses, and protists. TLR5, TLR2‐TLR1, TLR2‐TLR6, and TLR11 are located in the plasma membrane, whereas TLR3, TLR7‐TLR8, TLR9, and TLR13 are on endosomes 33, 34. TLR4 is present on both plasma membrane and endosomes. Stimulation of TLRs leads to their dimerization 33, 35. Although TLR3, TLR4, TLR5, TLR9, TLR11, and TLR13 form homodimers, TLR2 forms a heterodimer with TLR1 or TLR6, and TLR7 does so with TLR8. After ligand‐induced dimerization of TLRs, the adapter proteins myeloid differentiation primary‐response protein 88 (MyD88) and Toll‐interleukin (IL)‐1‐resistance domain‐containing adapter protein inducing IFNβ (TRIF) can be recruited to the receptors. All TLRs aside from TLR3 engage MyD88 upon activation, while TLR3 only recruits TRIF. Interestingly, TLR4 can recruit both MyD88 and TRIF, depending on its localization with TLR4 on the plasma membrane activating an MyD88‐dependent pathway and TLR4, which moves to endosomes switching to a TRIF‐dependent pathway 34.

MyD88 recruits IL‐1R‐associated kinase 1 (IRAK1) and IRAK2, IRAK4, TRAF3, TRAF6, and cIAPs 36. TRAF6 attaches Lys63‐linked polyubiquitin chain to cIAPs and to itself, creating a platform to recruit the IKK and TAB/TAK complexes, which activates NF‐κB and MAPK signaling pathways. In the TLR3 and TLR4 pathways, engagement of TRIF enables NF‐κB and MAPK signaling pathway activation and also production of type‐I interferons (IFNs). A complex formed by TRIF, TRADD, TRAF6, the E3 ligase Pellino‐1, and RIPK1 recruits the IKK and TAB/TAK complexes resulting in NF‐κB and MAPK activation 37, 38, whereas a complex composed of TRIF, TRAF3, tank‐binding kinase 1 (TBK1)/IKKε results in phosphorylation of the transcription factors interferon regulatory factor 3 (IRF3) and IRF7 and consequent type‐I IFN production 36.

LUBAC has been shown to be involved in TLR signaling. Primary B cells or macrophages of SHARPIN‐deficient mice display impaired NF‐κB activation after lipopolysaccharide (LPS)‐mediated TLR4 stimulation 12. The pro‐inflammatory cytokine induction in response to different TLR ligands in macrophages from SHARPIN‐deficient mice is also attenuated 39. In TLR1/2 signaling, IRAK1, IRAK4, and MyD88 have been suggested as substrates of LUBAC 40. For all other TLR signaling pathways, the targets of LUBAC have not yet been identified. Moreover, it is also still unclear how LUBAC is recruited to TLR signaling complexes. Thus, the role of linear ubiquitination in TLR signaling pathways requires further investigation.

Signaling through IL‐1 receptor (IL‐1R) shares the same molecules involved in TLR4 signaling. Binding of IL‐1 to IL‐1R recruits MyD88, IRAK1, IRAK4, TRAF6, and Pellino‐1 with consequent recruitment of the TAB/TAK and IKK complexes, allowing NF‐κB and MAPK activation 41. LUBAC depletion attenuates IL‐1‐mediated NF‐κB and MAPK signaling activation. Indeed, SHARPIN‐deficient primary keratinocytes and MEFs show defective IL‐1‐mediated NF‐κB and MAPK activation 11, 12, 13. Recently, Emmerich *et al*. 40 showed that linear ubiquitin chains, attached by LUBAC, are added onto existing Lys63‐linked Ub chains conjugated to IRAK1, and possibly to IRAK4 and MyD88, in the IL‐1 signaling.

### RLR signaling

The RLR family includes RIG‐I, the melanoma differentiation‐associated gene 5 (MDA5) and the laboratory of genetics and physiology 2 (LPG2) receptors. They are intracellular sensors of double‐stranded ribonucleic acid (dsRNA) and, as such, of actively replicating viruses. RIG‐I and MDA5 contain two caspase activation and recruitment domains (CARDs) at the N‐terminus, and a regulatory domain at the C‐terminus, a central DEAD‐box helicase/ATPase domain. LPG2 shares the same domains of RIG‐I and MDA5 with the exception of the CARD. RIG‐I and MDA5 sense the viral dsRNA via their helicase domain and recruit an adapter protein, mitochondrial antiviral signaling protein (MAVS), via their respective CARDs 42. Complete activation of the receptors requires the E3 ligase activity of the tripartite motif protein 25 (TRIM25) 43. Depending on the molecules recruited to the RIG‐I/MAVS complex, two different outputs can be achieved: NF‐κB and MAPK activation via the IKK and TAB/TAK complexes and the production of type‐I IFN via phosphorylation of IRF3/7 44. Downstream of RIG‐I, MAVS serves as a platform for recruitment of TRADD, caspase‐8, RIPK1, FADD, TRAF3, and TBK1/IKKε that promotes activation of IRF3 and IRF7 and consequent type‐I IFN production 44, 45, 46. When TRAF6 is recruited to the complex instead of TRAF3, together with TRAF2 and TRAF5 they are responsible for recruitment of the IKK and TAB/TAK complexes and NF‐κB and MAPK activation. However, recent findings show that TRAF6 can also be an IRF3 activator 47.

Recently, LUBAC has been implicated in the regulation of RIG‐I signaling 47, 48, 49. Surprisingly, however, LUBAC was reported to suppress RIG‐I‐mediated type‐I IFN production by inducing TRIM25 degradation and/or inhibiting the interaction between TRIM25 and RIG‐I. Consistent with that, depletion of either HOIL‐1 or HOIP enhanced RIG‐I‐mediated IFN production 49. Another study suggested that LUBAC downregulates RIG‐I‐mediated IFN production via linear ubiquitination of NEMO. According to their model, linearly ubiquitinated NEMO binds to TRAF3 and disrupts the TRAF3‐MAVS interaction, thereby reducing IFN production. Moreover, MEFs derived from SHARPIN‐deficient mice displayed high level of type‐I IFN production 48. In contrast with these studies, Liu *et al*. 47 showed that depletion of HOIP or SHARPIN decreases vesicular stomatitis virus‐mediated IFN secretion. Therefore, the role and mechanism by which LUBAC controls RIG‐I/MDA5 pathways is still controversial and needs further investigation.

### Inflammasome signaling

Inflammasomes comprise a family of cytosolic multi‐protein complexes that, in response to PAMPs and DAMPs, initiate an inflammatory response via activation of caspase‐1/11 and the consequent cleavage and secretion of IL‐1β and IL‐18. In addition, inflammasome activation can result in a type of inflammatory cell death, referred to as pyroptosis, which requires activity of caspase‐1 or ‐11 50, 51. Inflammasomes are activated by two types of cytoplasmic receptors appertaining to the NLR and ALR families. Inflammasomes are formed by pathogen‐sensing molecules, adapter proteins and caspase‐1. The NLR sensor molecules comprise the NOD‐, LRR‐, and pyrin domain‐containing 1 (NLRP1), NLRP3, NLRP6, NLRP7, NLRP12, or NOD‐, LRR‐, and CARD‐containing 4 (NLRC4, also known as IPAF). The NLR sensors, except for the NLRP1, contain three domains: a C‐terminal domain including several leucine‐rich repeats (LRRs), a central nucleotide‐binding domain NBD (also referred to as NACHT), and an N‐terminal effector domain, pyrin domain (PYD) on the NLRPs, and a CARD on NLRC4 52. In addition to these domains, NLRP1 contains a function‐to‐find domain that is required for its activation 53. The LRR domain has been implicated with the recognition of pathogens and DAMPs and auto‐regulation of NLRs. Nevertheless, it remains unclear whether the binding of molecules from pathogens to the sensors is direct or not. The NACHT domain is associated with oligomerization of the sensors and has an ATPase activity. The effector domain interacts with adapter proteins and with caspase‐1 directly.

Apoptosis‐associated speck‐like protein containing a caspase recruitment domain (ASC) is an adapter protein that interacts with inflammasome sensors via its PYD. Following this interaction, ASC dimerizes and, via its CARD, recruits monomers of pro‐caspase‐1 that are subsequently cleaved. Once activated, caspase‐1 cleaves pro‐IL‐1β and pro‐IL‐18 into their active forms. However, recent studies imply that ASC is dispensable for the NLRP1 inflammasome 54, 55. NLRC4 can activate caspase‐1 through its CARD without recruiting ASC, which is indeed not required for NLRC4‐mediated pyroptosis; nevertheless, the recruitment of ASC to the NLRC4 inflammasome is critical for processing of caspase‐1, IL‐1β, and IL‐18 54, 55.

The ALRs include two sensors able to induce inflammasome formation: the AIM2 and IFNγ‐inducible protein 16 (IFI16). AIM2 contains a PYD and a DNA‐binding HIN domain, whereas IFI16 has one PYD and two HIN domains for DNA binding. They are thought to directly bind to their ligands [viral double‐stranded deoxyribonucleic acid (dsDNA)] via the DNA‐binding HIN‐200 domain and to ASC via their PYD 56. Following ASC binding to the receptors, pro‐caspase‐1 is recruited to the inflammasome.

Dysregulation of inflammasomes has been associated with several auto‐inflammatory disorders and autoimmunity such as Crohn's disease, cryopyrin‐associated periodic syndromes, vitiligo and vitiligo‐associated Addison's disease, and familial Mediterranean fever 57. Inflammasome activation consists of two phases: priming and activation. In the priming phase, inflammasome components need to be expressed in an NF‐κB‐dependent manner. Subsequently, a specific inflammasome activator triggers formation of the inflammasome. Both phases are also regulated at the post‐translational level. For example, ubiquitination and deubiquitination has been reported to play an important role in the regulation of the NLRP3 inflammasome 58, 59, 60, 61.

Recent studies suggest that LUBAC is associated with particular inflammasomes. Specifically, SHARPIN and HOIL‐1 were shown to be required for NLRP3 inflammasome activation 60, 62. Rodgers *et al*. 60 showed that HOIL‐1‐deficient bone marrow‐derived macrophages (BMDMs) have reduced level of IL‐1β and caspase‐1 activation upon NLRP3 stimulation independently of NF‐κB activation. In contrast, HOIL‐1 deficiency has a minor impact on the NLRC4 and AIM2 inflammasomes. Moreover, ASC has been suggested as a substrate of LUBAC. HOIL‐1 is required for muramyldipeptide (MDP)‐induced peritonitis and for LPS‐induced sepsis. SHARPIN‐deficient BMDMs are defective in IL‐1β secretion and caspase‐1 activation upon several inflammasome ligands, especially upon stimulation of the NLRP3 inflammasome 62. It therefore appears that the involvement of SHARPIN in inflammasome activation is related to the NF‐κB‐mediated priming phase. This is in contrast with another report which concludes that regulation of the inflammasome by HOIL‐1 is NF‐κB‐independent 60. The role of HOIP in inflammasomes as the catalytic component of LUBAC still remains to be investigated. Thus, although it appears that LUBAC plays a role in inflammasome signaling, whether it is involved in the priming or activation phase requires clarification.

### NOD signaling

Other members of the NLR family, such as nucleotide‐binding oligomerization domain 1 (NOD1) and NOD2, instead induce NF‐κB and MAPK activation upon stimulation with peptidoglycans derived from bacterial cell walls. NOD1 specifically recognizes D‐glutamyl‐meso‐diaminopimelic acid (iE‐DAP) and NOD2 recognizes MDP. Stimulation of NOD1 or NOD2 leads to formation of the NOD signaling complex (NOD‐SC) that contains, aside from the respective receptor, also RIPK2, TRAF2, XIAP, and cIAP1/2 63. It has been reported that cIAP1/2 and XIAP attach Lys63‐linked polyubiquitin chains to RIPK2 64, 65. This mediates recruitment of the IKK and TAB/TAK complexes to enable activation of NF‐κB and MAPKs. LUBAC forms part of a NOD2‐SC that forms upon NOD2 overexpression and its recruitment to overexpressed NOD2 depends on the E3 activity of XIAP. Without LUBAC, NF‐κB activation upon NOD2 stimulation is strongly inhibited 65, 66. A substrate of LUBAC in NOD2 signaling is RIPK2 67.

## LUBAC in adaptive immune signaling

The adaptive immune system is responsible for a second line of defense against pathogens which is a more specific and sustained immune response. Adaptive immunity is mediated by T and B cells. B cells are activated via direct contact with antigen through their B‐cell receptor (BCR). Once activated, B cells proliferate, mature into plasma cells, and secrete antibodies. They are the principal mediator of humoral (or antibody‐mediated) immunity. Cell‐mediated adaptive immunity, in turn, is carried out by T cells with the support of dendritic cells, macrophages, and natural killer cells 31.

The CD40/CD40L signaling pathway is crucial for effective T‐ and B‐cell immune responses. CD40L expressed on T cells and other cells in inflammatory conditions binds to its receptor CD40, a member of the TNFR superfamily, on B cells. The CD40 signaling pathway activates NF‐κB signaling. Binding of CD40L to CD40 leads to formation of a protein complex composed of TRAFs and cIAPs that in turn recruits the IKK and the TAB/TAK complexes to enable NF‐κB and MAPK activation 68. LUBAC forms part of the CD40 complex and its recruitment depends on the E3 activity of cIAPs 11. B cells devoid of LUBAC components have impaired CD40‐mediated NF‐κB activation 11, 69. Moreover, mice lacking HOIP activity specifically in B cells (B‐HOIP Δlinear) display defective B‐cell development in the peritoneal cavity but not in the bone marrow 70. Activation of NF‐κB and ERK by CD40, LPS, or transmembrane activator, and calcium modulator, and cyclophilin ligand interactor (TACI) is impaired in B cells from B‐HOIP Δlinear mice. The immune response to thymus‐dependent antigens is also impaired in B cells lacking HOIP activity. Although the target of LUBAC in CD40 signaling has not been identified, these results indicate that LUBAC activity is important for proper CD40 signaling (*Table* 1).

**Table 1:** Phenotypes of mice with deficiency or mutation in linear ubiquitin chain assembly complex (LUBAC) components or in OTU DUB with linear linkage specificity (OTULIN)

Genotype	Phenotype	Not affected by	Partially rescued by	Completely rescued by
*Sharpin* ^*cpdm*^	Chronic proliferative dermatitis/multiorgan inflammation/splenomegaly and loss of Peyer's patches 84, 85	*Tnfr2* ^*−/−*^ 86	*Il1r1* ^*−/−*^ 58	*Tnfr1* ^*−/−*^ 86
		*Bid* ^*−/−*^ 58	*Caspase‐8* ^*+/−*^ 86	*Ripk3* ^*−/−*^ *; Caspase‐8* ^*+/−*^ 86
		*Rag1* ^*−/−*^ 85	*Ripk3* ^*−/−*^ 86, 91	*Ripk3* ^*−/−*^ *; Fadd* ^*E‐KO*^ 91
		*Il‐5* ^*−/−*^ 89	*Mlkl* ^*−/−*^ 86	
			*Tradd* ^*E‐KO*^ 91	
			*Tnf* ^*−/−*^ 11	
			*Tnfr1* ^*E‐KO*^ 91	
			*Tnfr1* ^*−/−*^ 86	
*Hoip* ^*−/−*^	Lethality at E10.5/vasculature defects 30	n.d.	*Tnf* ^*−/−*^ up to E15.5 30	n.d.
			*Tnfr1* ^*−/−*^ up to E17.5 30	
*Hoip* ^*C879S*^	Lethality at E11.5 40	n.d.	n.d.	n.d.
B‐HOIP Δlinear	Defect in B1 development/impaired CD40‐mediated NF‐*κ*B and MAPK 70	n.d.	n.d.	n.d.
*Hoil‐1* ^*−/−*^	No overt phenotype 13	–	–	–
*Otulin* ^*gumby*^	Lethality at E12.5‐14.5/vasculature defects 102	n.d.	n.d.	n.d.

Signaling via the BCR is central for the activation and function of B cells. Unexpectedly, while HOIP is required for BCR signaling, HOIP activity was found to be dispensable for BCR‐mediated NF‐κB activation 70. Two single‐nucleotide polymorphisms (SNPs) in the HOIP gene have an overall frequency of 7.77% in activated B‐cell like (ABC) diffuse large B‐cell lymphoma (DLBCL) patients 71. Both SNPs are located in the UBA domain of HOIP which is essential for HOIL‐1 binding. These mutations result in increased NF‐κB activation and enhanced HOIP‐HOIL‐1 interaction. On the other hand, ABC cell lines treated with a peptide that inhibits the HOIP‐HOIL‐1 interaction display decreased NF‐κB activation and cell viability. In support of this, HOIP knockdown reduces NF‐κB activation and cell viability in ABC DLBCL lines 72. Moreover, the authors provide evidence that LUBAC is also implicated in T‐cell receptor (TCR)‐mediated NF‐κB activation as knockdown of HOIP or SHARPIN decreased TCR‐mediated NF‐κB activation. Surprisingly, expression of inactive HOIP did not, however, influence NF‐κB activation, implying that the role of LUBAC in TCR‐mediated NF‐κB activation is independent of the E3 ligase activity of HOIP.

## Cell death and sterile inflammation

**Figure 2:**
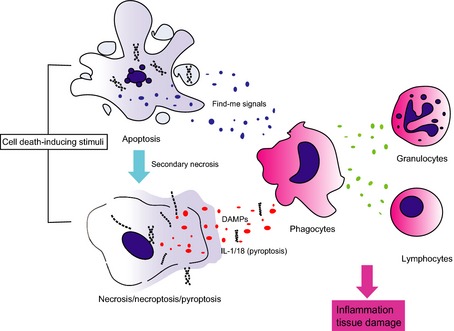
**Cell death induces inflammation.** Several external stimuli can trigger different types of cell death. Necrosis and pyroptosis compromise plasma membrane integrity, which leads to the release of damage‐ (or danger‐) associated molecular patterns (DAMPs). Although apoptotic cells normally release limited DAMPs, they can produce cytokines and chemokines (so‐called find me signals), which can also attract immune cells. However, when apoptotic cells are not engulfed by phagocytes in a timely manner, which can occur when apoptosis induction is too massive or too rapid, they undergo secondary necrosis, and can hence also release DAMPs. These activate inflammatory cells which in turn secrete factors that attract and activate other types of immune cells. In summary, cell death can induce an inflammatory response which can result in tissue damage, hence autoinflammation and possibly also autoimmunity.

Trauma and toxin exposure can cause cell death in tissues which could be causative for sterile inflammation. Dying and dead cells expose and release multiple proteins and substances, that are usually contained when the cells are alive, which can attract and activate immune cells. The recognition of PAMPs plays a vital role in the detection of dead cells, similar to the detection of infectious agents 73. For instance, autologous nuclear DNA and mitochondrial DNA are recognized by innate immune cell‐expressed TLR9. As mentioned before, in addition to PAMPs, several DAMPs alert the presence of dead cells to immune cells. They include high mobility group box 1 protein, heat‐shock proteins, IL‐33, uric acid crystals, and adenosine triphosphate/uridine triphosphate (ATP/UTP) 74. The release of DAMPs is thought to be a common feature of necrotic cell death 75. DAMPs also activate PRRs that can bind to PAMPs (*Fig*. 2).

In contrast to necrosis, during apoptosis, cells maintain plasma membrane integrity, and do not release intracellular content or DAMPs until late in the process 75. Apoptotic cells alter their membrane composition from their viable state so that they can be recognized by phagocytes. Indeed, the engulfment of apoptotic cells by phagocytes promotes the secretion of anti‐inflammatory chemokines and cytokines to dampen inflammation 76. Apoptosis is thus thought to be more immunologically inert than necrosis. However, recent studies shed light on the active side of apoptosis with regard to inflammation. For instance, CD95 (Fas)‐induced cell death coincides with the production of chemokines and cytokines and to attract immune cells 77. In the case of conditional deletion of *Tak1* in the liver, which causes inflammation‐associated carcinogenesis, concurrent ablation of caspase‐8 results in reduced cell death, amelioration of inflammation, and prevention of liver carcinogenesis 78. Another example is the specific deletion of *Ripk1* in intestinal epithelial cells (*Ripk1* ^iEC‐KO^), which results in spontaneous severe gut inflammation and lethality within 4 weeks of age 79, 80. Conditional ablation of apoptosis through genetic deletion of *Fadd or Caspase‐8*, but not *Ripk3*, in intestinal epithelial cells completely prevents cell death and rescues the phenotype of *Ripk1* ^*iEC‐KO*^ mice 79, 80. Deletion of *Ripk1* in the epidermal keratinocytes (*Ripk1* ^*E‐KO*^) instead leads to a skin inflammatory phenotype that is completely prevented by genetic ablation of the two known downstream components of necroptosis signaling, RIPK3 or MLKL, indicating that in this case aberrant necroptosis is causative for inflammation 79. Mice with specific deletion of *Fadd* or *Caspase‐8* in epidermal keratinocytes also develop severe skin inflammation that is abrogated by concomitant *Ripk3* deletion, implying that increased necroptosis triggers skin inflammation 79. Therefore, exacerbated cell death, which can either be apoptotic or necroptotic, can result in an inflammatory phenotype (*Fig.* 2).

In humans, cell death is involved in multiple human diseases, such as sepsis, ischemia‐reperfusion injury, liver disease, and cancer. Because cell death and inflammation can trigger each other, exacerbated cell death further amplifies inflammation, and *vice versa*, in tissues 81. Tight regulation of cell death is therefore needed to prevent inflammatory disease.

## LUBAC deficiency in mice and humans

As discussed above, LUBAC is crucial to maintain the balance between gene‐activatory and cell death signaling pathways upon different stimuli. Therefore, absence or attenuation of LUBAC activity leads to aberrant signaling output with decreased gene activation and increased cell death. Here, we discuss the effect of deficiency of the three different LUBAC components in mice and humans which, depending on the component that is absent, results in developmental defects, immunosuppression, inflammation, and/or autoimmunity.

### Sharpin‐deficient mice

Mice with a spontaneous deletion in the first exon of the *Sharpin* gene, which results in complete absence of expression of SHARPIN protein, develop chronic proliferative dermatitis (referred to as *cpdm*) at 3–6 weeks of age 82, 83. Increased cell death indicators, cleaved caspase‐3, ‐7, and ‐9, are detected as early as at 2 weeks of age in the epidermis of *cpdm* mice 12, 84, 85, 86. Primary keratinocytes and MEFs from *cpdm* mice are more sensitive to TNF‐induced death and have a decreased capacity to activate NF‐κB in response to TNF 11, 12, 13. The skin of *cpdm* mice is characterized by epidermal hyperplasia, hyperkeratosis and parakeratosis and it is highly inflamed with accumulation of eosinophils, macrophages, and neutrophils. It has an increased expression of type 2 cytokines, which is a feature of type 2 inflammatory responses 87. Inflammation in other organs such as the liver, gut, lung, and esophagus is also observed in these mice, together with splenomegaly and loss of Peyer's patches 82, 88.

To understand the contribution of hematopoiesis to the pathogenesis of *cpdm* mice, several genetic experiments have been performed. *Cpdm* mice lacking B and T cells (*cpdm; Rag1* ^*−/−*^) still develop dermatitis, indicating that the lymphoid compartment is dispensable for development of the *cpdm* phenotype 85. While depletion of eosinophils, either via IL‐5‐neutralizing antibodies or by genetic ablation of IL‐5, effectively decreased the number of cutaneous and circulating eosinophils, dermatitis was not ameliorated, demonstrating that eosinophils do not play a vital role in the development of the *cpdm* pathology 89. Also, transfer of bone marrow cells and splenocytes from *cpdm* to wildtype mice was incapable of transferring the disease 82, 86, 90. On the other hand, *cpdm* skin transplantation onto nude mice maintained the donor phenotype 3 months after transplantation 90. Together, these results point toward the *cpdm* phenotype likely being a result of a skin‐intrinsic defect and that the hematopoietic compartment is unlikely to play a crucial role in the development of the inflammatory manifestations of the *cpdm* phenotype.

As we found increased cell death in the epidermis of *cpdm* mice and that cpdm‐derived keratinocytes underwent aberrant TNF‐induced death *in vitro*, we ablated TNF in *cpdm* mice which resulted in complete prevention of inflammation 11. Together, this allowed us to conclude that TNF‐mediated cell death was responsible for the inflammatory manifestations of the *cpdm* phenotype. This conclusion was confirmed in two recent studies, which in addition addressed the modality of TNF‐induced death that is causative for inflammation in *cpdm* mice. Consistent with TNF‐induced killing of keratinocytes via TNFR1 being responsible for the observed inflammation, constitutive ablation of TNFR1 but not TNFR2 86, and deletion of TNFR1 specifically in the epidermal keratinocytes (*Tnfr1* ^*E‐KO*^) 91 prevented development of skin lesions in *cpdm* mice. Address the role necroptosis in the pathogenesis of *cpdm* mice, ablation of RIPK3 slightly delayed the appearance of the skin lesion in *cpdm* mice, whereas ablation of MLKL did not influence dermatitis in these mice. Interestingly however, co‐deletion of *Ripk3* or *Mlkl* significantly ameliorated cell death and inflammation in other organs 86, 91. The differential effects of ablation of RIPK3 and/or MLKL on the skin phenotype of *cpdm* mice imply that RIPK3 contributes to the *cpdm* skin phenotype in a manner that is independent of MLKL‐mediated necroptosis. Therefore, while RIPK3‐mediated MLKL‐dependent necroptosis is unlikely to be the sole driver of inflammation in the skin of *cpdm* mice, RIPK3‐mediated MLKL‐dependent and ‐independent effector mechanisms appear to synergize in the development of inflammation in other organs of *cpdm* mice. Caspase‐8 heterozygosity, but not BID deficiency, significantly delayed the onset of skin lesions in *cpdm* mice. The complete rescue of the *cpdm* phenotype is observed with *Ripk3* deletion and heterozygous knockout of caspase‐8 (*cpdm; Ripk3* ^*−/−*^ *; Caspase‐8* ^*+/−*^) 86 as well as by the combination of *Fadd* ^*E‐KO*^ and *Ripk3* ^*−/−*^ 91 (*Table* 1). We can therefore conclude that TNF‐mediated cell death, mainly by apoptosis, appears to be causative for the inflammatory manifestations of the *cpdm* phenotype.

Surprisingly however, co‐deletion of *Ripk3* and both alleles of the *Caspase‐8* gene rendered *cpdm* mice lethal between embryonic day (E) 17 and 3 months of age. The reason for the lethality of triple deficiency in SHARPIN, RIPK3, and caspase‐8 remains unresolved. In this context it is noteworthy to mention that expression of a kinase inactive version of RIPK1 (*Ripk1* ^*K45A*^) instead of wildtype RIPK1 completely rescues the *cpdm* phenotype 92. It therefore appears that the lethality of the *cpdm; Ripk3* ^*−/−*^ *; Caspase‐8* ^*−/−*^ is mediated by an uncontrolled kinase activity of RIPK1, unleashed by deficiency in SHARPIN when both RIPK3 and Caspase‐8 are also not present, as RIPK3/Caspase‐8‐double‐knockout mice do not show any such defects.

### HOIP‐deficient mice

Deficiency in HOIP (*Hoip* ^*−/−*^) results in embryonic lethality at E10.5 caused by defective vascularization in the yolk sac. This is phenocopied by HOIP deficiency specifically in endothelial cells 30. Cell death, characterized by positivity in TUNEL and/or cleaved caspase‐3 staining, rises in the yolk sac of systemic and endothelial‐specific HOIP‐deficient embryos prior to their death at E10.5. The exacerbated cell death is mediated by both, TNF and LTα as genetic ablation of TNF delayed lethality of the *Hoip* ^*−/−*^ to E15.5 and that of TNFR1 prolonged their survival until E17.5, with a significant decrease in cell death in the yolk sac and a complete rescue of the vascularization defects in the latter case (*Table* 1). Nevertheless, *Tnfr1* ^*−/−*^ *Hoip* ^*−/−*^ embryos do not survive after late gestation 30. The reason for this lethality is not yet known, and it will be interesting to determine the cause of death of these mice at E17.5. Intriguingly, cIAP1/2‐double‐deficient mice are rescued by TNFR1 deficiency, at least up until birth 93. It can therefore be concluded that the HOIP‐regulated pathway responsible for lethality of these mice is cIAP1/2‐independent.

It appears that mice expressing catalytically inactive HOIP instead of wildtype HOIP also die at an early embryonic stage 40, 70. However, a detailed biochemical and genetic analysis of such mice has not been presented (*Table* 1).

### HOIL‐1‐deficient mice and patients

Mice deficient for HOIL‐1 are viable and do not appear to present with any overt phenotype 94 (*Table* 1). Yet, administration of TNF causes liver injury in *Hoil‐1* ^*−/−*^ mice. MEFs and primary hepatocytes obtained from *Hoil‐1* ^*−/−*^ mice are defective in TNF‐induced NF‐κB activation 94. Despite this rather unspectacular phenotype in mice, human patients with loss‐of‐expression and loss‐of‐function mutations in HOIL‐1 develop chronic autoinflammation, pyogenic bacterial disease and muscular amylopectinosis, resulting in their premature death during infancy. Although fibroblasts from these patients are impaired in TNF‐ and IL‐1β‐dependent NF‐κB activation, their leukocytes are hyper‐responsive to IL‐1β 95. Hence, different cell types appear to respond differently to distinct pro‐inflammatory stimuli, which could explain the co‐presence of auto‐inflammation and immunodeficiency in the patients. Interestingly, anti‐TNF treatment was capable of reducing clinical inflammation in one of the *HOIL‐1* ^*−/−*^ patients, in line with the effect of TNF deletion in the *cpdm* mice 95.

## Deubiquitinases for linear ubiquitin chains

DUBs counteract ubiquitination by removing ubiquitin from substrates. They are also responsible for processing the ubiquitin precursor after translation. Nearly 100 DUBs are encoded by the human genome, and they are stratified into five families according to their specific DUB domains. There are ubiquitin carboxy‐terminal hydrolases, ubiquitin‐specific proteases (USPs), Machado–Joseph disease protein domain proteases, ovarian tumor proteases (OTUs), and JAB/MPN/Mov34 metalloenzyme domain proteases 96. The function of the DUB is determined by which ubiquitin chain they remove. One of the well‐known functions of DUBs is to rescue proteins from proteasomal degradation by removing Lys48‐linkages attached to them. Yet, the result of a DUB on non‐degradative ubiquitin modifications, including on Met1‐mediated linkages, is at present still unclear. Intriguingly, the two DUBs that have so far been proposed as capable of cleaving Met1‐linked ubiquitin, CYLD, and OTU DUB with linear linkage specificity (OTULIN), have recently been shown to occur in complex together with LUBAC as both proteins interact with the PUB domain of HOIP 97, 98, 99. OTULIN is a highly specific DUB for the linear ubiquitin linkage, whereas CYLD can cleave linear Ub but also Lys63‐Ub 100, 101.

### OTULIN

OTULIN, also known as FAM105B, is a member of the OTU family and shares a similar structure to that of OTUB1 100, 102. While the other 14 annotated OTU DUBs cannot hydrolyze Met1‐di‐Ub, OTULIN exclusively cleaves Met1‐Ub 100, 102. OTULIN is approximately 100‐fold more specific for cleaving Met1‐di‐Ub than Lys63‐di‐Ub, although Met1‐Ub and Lys63‐Ub are structurally quite similar as both adopt an extended conformation 100. The specificity of OTULIN toward Met1‐di‐Ub is attributed to its S1’ binding site. The binding of the proximal Ub to the S1’ site fixes it at the position where all other lysine residues in the Ub moiety, except for Lys63, are distant from the catalytic center. Importantly, Lys63 is covered by a dedicated pocket so that OTULIN can distinguish Met1 from Lys63 despite the two of them being spatially close to each other. The requirement of glutamate (Glu) 16 of the proximal Ub for the cleavage activity of OTULIN suggests the presence of Ub‐mediated substrate‐assisted catalysis 100.

OTULIN interacts with HOIP via its PUB‐interacting motif (PIM), which binds to the N‐terminal PUB domain of HOIP. Phosphorylation of OTULIN's tyrosine (Tyr) 56 prevents the binding of OTULIN to HOIP 97, 98. It is currently unknown whether this phosphorylation plays a role in regulating LUBAC activity, and it is also currently unknown which kinase is responsible for this phosphorylation. Consistent with a role for OTULIN in counteracting linear ubiquitination, its overexpression was shown to delay LUBAC‐mediated NF‐κB signaling and JNK activation upon TNF, whereas its inducible knockdown was reported to increase these signaling outputs 67, 100. Unexplained, however, is the observation that overexpression and knockdown of OTULIN both sensitize cells to TNF‐induced cell death 100. This observation suggests that an optimal level of linear ubiquitination might be required for cell survival and that both too much or too little could be equally detrimental.

Interestingly, *gumby* mice carry a mutation in the gene encoding *Otulin/Fam105b* that is causative for reduced craniofacial vasculature from E10.5 and embryonic lethality between E12.5‐E14 102 (*Table* 1). *Gumby* mice exhibit abnormal vessel branching in the head and trunk, although the major vessels appear to develop normally. Although the cause of death of OTULIN‐mutant embryos appears to be different from that of HOIP‐deficient mice, it is peculiar that both types of mice die due to deficits in vascularization 30, 102. Yet, as OTULIN‐mutant embryos die approximately 2 days later than HOIP‐deficient embryos 30, 102, there might be differences that underlie the mechanisms of death of OTULIN‐ versus HOIP‐mutant embryos. The observations that OTULIN mutation negatively impacts vessel development and that OTULIN interacts with dishevelled (DVL) 2, a Wnt signaling adapter, suggested that linear ubiquitination could be involved in Wnt signaling. In line with this, LUBAC overexpression negatively regulates canonical Wnt signaling while OTULIN overexpression promoted it 102. The molecular mechanism, however, as to how LUBAC and OTULIN would control canonical Wnt signaling remains largely elusive.

### CYLD

CYLD is a tumor suppressor belonging to the USP family of DUBs. CYLD is thought to specifically cleave Lys63‐ and Met1‐linkages 101, 103, 104, although one study suggested that CYLD can also cleave other di‐Ub linkages 105. A recent structural study revealed that the dual specificity is supported by its flexible binding domain to the proximal ubiquitin. CYLD can accommodate both linkage types in a specific manner although they are in a different orientation 104.

Mutations in the gene encoding CYLD are causative for familial cylindromatosis 106. Most *CYLD* mutations are clustered near the catalytic USP domain at the C‐terminus. Interestingly, the USP domain of CYLD has only marginal homology to the other members of the USP family. This is due to a B‐box domain insertion right in the center of the USP domain 103. Loss of CYLD leads to an aberrant increase in inflammatory signaling notably increased activation of NF‐κB 107. This has also been shown to increase the formation of inflammation‐associated cancers including colorectal, skin, and liver cancers 108, 109, 110. In addition, loss or downregulation of CYLD has been observed in several human cancers including of the colon, the liver 111 and the skin 106.

CYLD activity is regulated by cleavage and phosphorylation. CYLD plays a role in various immune signaling pathways and multiple proteins that are crucial for these pathways have been identified or proposed as targets of CYLD's DUB activity, including TRAF2/6, RIPK1, NEMO, TAK1, and RIG‐I 107. At present, CYLD is thought to remove Lys63‐Ub from these targets proteins and to thereby shut off signaling. It will be interesting to determine whether CYLD may also act on Met1‐Ub on these or other targets contained in the central signaling complexes of these immune signaling pathways. CYLD is also important for cell death induction via TNFR1 as it has been shown to be required for the induction of necroptosis by facilitating the formation of complex II of TNFR1 signaling. The pro‐apoptotic complex II component caspase‐8 can cleave CYLD which results in its degradation and prevention of CYLD‐dependent necroptosis 22. In addition to proteolytic cleavage, CYLD activity is also negatively controlled by phosphorylation. Downstream of TNFR1, CYLD has been proposed to be phosphorylated by IKKα/β in a NEMO‐dependent manner and that this enables TRAF2 to be sufficiently ubiquitinated to signal optimally 112.

CYLD has also been reported to act as a negative regulator of canonical Wnt signaling by removing Lys63‐chains from DVL2 113. CYLD levels are decreased in human tumors, which correlates with upregulated Wnt signaling as characterized by β‐catenin accumulation and Axin‐2 expression 113. Consistent with this, upregulation of β‐catenin is also observed in CYLD‐deficient mouse livers 109. In addition, a mutant version of HOIP that can neither bind to OTULIN nor CYLD is also less capable of inducing canonical Wnt signaling than wildtype HOIP 99.

## Linear ubiquitin‐binding proteins

It recently emerged that most proteins with ubiquitin‐binding domains interact specifically with particular types of Ub linkages. Thereby certain functional units are recruited to specific Ub chains on particular targets within immune receptor signaling complexes. Although some types of linkages create structurally similar di‐Ub, notably Met1‐linked and Lys63‐linked di‐Ub, there are a clear distinction between them in cells. The NZF domain of TAB2 and the ubiquitin binding in ABIN and NEMO (UBAN) motif of NEMO have specific affinities for Lys63‐ and Met1‐linked di‐Ub linkages, respectively 114, 115, 116. For the UBAN motif of NEMO, this distinction is possible because it simultaneously interacts with sites in the proximal and distal ubiquitin of Met1‐linked di‐Ub, whereas it can only interact with one of them in Lys63‐linked di‐Ub 116, 117.

In the next sections, we discuss the three linear ubiquitin binding proteins known so far to regulate immune signaling pathways, i.e. NEMO, A20, and the A20‐binding and inhibitor of NF‐κB‐1 (ABIN‐1). Importantly, the functions of these proteins largely depend on the association with ubiquitin. Disruption of their ubiquitin binding capacity in rodent models results in pathophysiological features which are closely related to immune disorders in humans. Thus, their preferred affinity to linear ubiquitin further highlights the significance of linear ubiquitin in regulating immunity.

### NEMO

NEMO, encoded by an X‐linked gene (*IKBKG*) in both humans and mice, forms the IKK complex together with the two kinases IKKα and IKKβ to activate canonical NF‐κB signaling 118. Mutations in the gene encoding NEMO are associated with X‐linked genetic diseases in humans, including incontinentia pigmenti 119 and anhidrotic ectodermal dysplasia with immunodeficiency (EDA‐ID) 120. As mentioned above, NEMO contains a ubiquitin recognition motif, referred to as the UBAN motif. The UBAN motif is located at the center of the coiled coil 2 (CC2) and leucine zipper (LZ) region (CC2‐LZ or CoZi domain) at the C‐terminus of NEMO. The UBAN motif has a specific binding capacity (*K*_*d*_ = 1.6 μM) for linear di‐Ub compared to Lys48‐ and Lys63‐linked di‐Ub 117. The CoZi domain dimerizes and binds to di‐Ub at a 2:1 ratio 117, 121. A point mutation in the UBAN motif, aspartate (D) 311 to glutamine (N) (D311N) of murine NEMO is sufficient to prevent IKK activation in NEMO‐deficient cells as it is unable to bind to Lys63‐ or Met1‐linked polyubiquitin 117, 121. Furthermore, mutations in the UBAN domain of NEMO are linked to EDA‐ID 120, 122. Taken together, these studies support the notion that the recognition of Met1‐linked ubiquitin by NEMO is necessary for optimal NF‐κB activation.

In addition to its binding capacity to linear ubiquitin, NEMO itself can be a substrate of LUBAC. Two ubiquitination sites, Lys285 and Lys309, have been reported to be important for the activation of NF‐κB upon LUBAC overexpression and IL‐1 stimulation 94. Importantly, a non‐ubiquitinatable mutant of NEMO (K285R/K392R) largely phenocopies loss of NEMO. Female mice heterozygous for the K285R/K392R mutation exhibit TNFR1‐dependent inflammatory skin lesions and splenomegaly 123, similar to female mice heterozygous for NEMO deficiency. In contrast, NEMO‐K392R mutant mice display no overt phenotype 124. Together, these studies suggest that Lys285 of NEMO, which appears to be linearly ubiquitinated, is the crucial ubiquitination site to regulate NF‐κB signaling.

### A20

A20 (also known as TNFAIP3) contains an OTU domain at the N‐terminus and seven ZF domains at the C‐terminus 125. A20 is thought to act as a DUB via its OTU domain and, at the same time, as an E3 ligase with its zinc‐finger domains to ubiquitinate substrates with Lys48‐linked polyubiquitin to degrade them 126. Yet, the specificity of A20's DUB activity is controversial. A20 was initially thought to serve as a suppressor of NF‐κB by cleaving Lys63‐di‐Ub linkages 127. However, other studies indicated that A20 is only capable of cleaving Lys11‐ and Lys48‐linked di‐Ub 105, 128. Moreover, A20 is incapable of hydrolyzing Lys63‐linked di‐Ub linkages on TRAF6, but rather targets the TRAF6‐ubiquitin junction to remove all of the ubiquitin modification *en bloc* 129.

A20 restricts activation of NF‐κB downstream of TNFR1, CD40, TLRs, IL‐1R, and NODs 127, 130, 131, 132. Mice deficient for A20 develop MyD88‐dependent severe multi‐organ inflammation and cachexia and die within 2 weeks of birth 127, 133. A20‐deficient cells show TNF‐induced NF‐κB hyperactivation but, counter‐intuitively, also increased cell death. Surprisingly, inactivation of A20's DUB activity by mutating Cys103 to alanine (A) (C103A) in the OTU domain does not elicit spontaneous inflammation in mice 134. The C103A mutant only marginally impacts NF‐κB activation in macrophages and dendritic cells 134. This suggests that the DUB activity of A20 is in fact dispensable for the negative control of NF‐κB activation. This inevitably brings up a question: which function of A20 is crucial for the regulation of NF‐κB signaling, and, thereby, inflammation?

A20 has two ubiquitin binding sites in its C‐terminus, ZF4 and ZF7, and both support the E3 ligase function of A20 125. ZF4 is the binding site to Lys63‐linked di‐Ub, but not to Lys48‐ or Met1‐linked di‐Ub. Mutation of ZF4 in mice does not affect the anti‐inflammatory function of A20 as mice harboring this mutation develop normally 135. ZF7, in contrast, is the binding site for Met1‐linked Ub and mediates A20 recruitment to the TNF‐RSC 136. ZF7 of A20 directly inhibits the TAK1‐dependent activation of NEMO by binding to ubiquitin chains 136, 137, 138. Moreover, this inhibition is independent of A20's catalytic activity as a DUB 136, 137, 138. A genetic mutation in ZF7 is known to be causative for B‐cell lymphoma in humans 136, 138. It is therefore tempting to postulate that the interaction of ZF7 of A20 with linear ubiquitin is crucial for the regulation of multiple immune signaling pathways. To test this, it would be interesting to investigate the anti‐inflammatory role of A20's ZF7 in mice with a mutation in ZF7 that interferes with Met1‐di‐Ub binding, as recently suggested by Verhelst *et al*. 126.

A20 also serves as an inhibitor of NLRP3 inflammasome activation/activity although not of the AIM2 and NLRC4 inflammasomes 139, 140. A20 restricts NLRP3‐mediated caspase‐1 activation and priming of NLRP3 and pro‐IL‐1β/IL‐18 by downregulating NF‐κB signaling upon TLR stimulation. A20 is in a complex with pro‐IL‐1β, caspase‐1, caspase‐8, RIPK1, and RIPK3 139. Lys63‐linked and unanchored ubiquitin chain appears to form part of this complex, but the presence of Met1‐linked ubiquitin chains has not yet been addressed. A20 also plays crucial roles in immune homeostasis in different immune cell compartments. Specific ablation of A20 in B cells results in abnormal B‐cell development and differentiation. A20‐deficient B cells display hyper‐responsiveness, such as enhanced proliferation and higher expression of the B‐cell activation markers, together with increased activation of canonical NF‐κB in response to B‐cell mitogens 130, 141. Mice with A20‐deficient B cells exhibit splenomegaly, plasma cell hyperplasia, and autoimmunity when aged 130, 141. Myeloid‐specific ablation of A20 in mice induces development of spontaneous destructive arthritis 142. This also causes high serum levels of inflammatory cytokines related to rheumatoid arthritis (RA) in humans. Although ablation of TNFR1 signaling did not ameliorate the RA‐like phenotype, intriguingly, depletion of MyD88, and neutralization of LPS by soluble LPS‐receptor mitigated inflammation. Thus, the TLR4‐MyD88 pathway, rather than TNF‐TNFR1 signaling, turns out to be critical for development of the RA‐like phenotype in this model 140, 142. In addition, depletion of A20 from dendritic cells resulted in the development of colitis 143 and systemic lupus erythematosus (SLE)‐like autoimmunity 144. Multiple SNPs in the *A20* gene are indeed associated with multiple immune diseases including SLE in humans as well 145, 146.

### ABIN‐1

ABIN‐1 was originally identified as an A20‐interacting protein in a yeast two‐hybrid screen 147. Similar to NEMO, ABIN‐1 has a UBAN motif at its C‐terminus 66. The UBAN motif of ABIN‐1 also preferentially binds Met1‐ over Lys63‐linked di‐Ub. ABIN‐1 suppresses NF‐κB activation during TNFR1 and TLR signaling by binding to NEMO and cooperating with A20 148. ABIN‐1 restricts TNF‐induced cell death by binding to ubiquitin chains 149.

Constitutive ablation of ABIN‐1 in mice causes lethality late during embryonic development and spontaneous autoimmunity and inflammation in rarely born mice. This lethality is neutralized by concomitant TNF ablation 149. The D485N mutant of murine ABIN‐1, which is equivalent to the D311N, UBAN motif mutant of murine NEMO, loses binding capacity to both Met1‐ and Lys63‐linked di‐Ub 150 and fails to protect against TNF‐induced cell death 149. ABIN‐1‐D485N mutant mice spontaneously develop several immune defects: splenomegaly, lymphadenopathy, germinal center formation in the spleen, and autoimmunity. Interestingly, these immune defects are MyD88‐dependent, as MyD88 deficiency corrects all these abnormalities 150. Nonetheless, expression of the ABIN‐1‐D485N instead of wildtype ABIN‐1 does not result in embryonic lethality. Therefore, other regions of ABIN‐1, aside from its UBAN motif, appear to contribute to its pro‐survival role in TNFR1 signaling *in vivo*.

ABIN‐1 is associated with psoriasis and systemic lupus erythematosus in humans 151, 152. Deletion of ABIN‐1 from dendritic cells disrupts the immune homeostasis and induces splenomegaly and lymphadenopathy. ABIN‐1‐deficient dendritic cells display hyper‐activation of NF‐κB and MAPK signaling and hyper‐production of IL‐23 in response to TLR ligands. Interestingly, ABIN‐1 protects against TLR7 ligand‐induced psoriasis by MyD88‐mediated signaling in dendritic cells 153.

## Identification of novel targets of linear ubiquitination

Despite the importance to distinguish diverse inter‐ubiquitin linkage types in biological systems, it has been challenging to pinpoint specific linkage types, including linear ubiquitination, due to their relatively low abundance and chemical similarity in nature 154. Several attempts have been made to develop tools to probe various types of ubiquitin linkages *in situ*: (i) linkage‐specific antibodies; (ii) tools to concentrate ubiquitinated proteins such as anti‐di‐glycyl lysine antibodies and ubiquitin sensors; (iii) sensitive mass‐spectrometric analysis; and (iv) recombinant DUBs.

First, linkage‐specific antibodies can detect the presence of specific ubiquitin linkages. These antibodies were raised by immunization with synthetic peptides that are similar to the specific link 94, or by phage‐display methods 155. At present, several specific Met1‐di‐Ub‐linkage‐specific antibodies are commercially available. After immunoprecipitating the protein of interest, Met1‐di‐Ub‐specific antibodies can be used to read out the presence of Met1‐linked ubiquitin on the protein by Western blotting. However, to be sure that the protein is indeed linearly ubiquitinated and to assess to which extent this is the case, specificity of the detected linkage has to be confirmed, a task that can be achieved by the use of recombinant DUBs that can cleave linear ubiquitin chains as compared to other types of Ub linkages (see below).

Second, different tools such as anti‐di‐glycyl lysine antibodies and ubiquitin sensors have been developed to enrich for ubiquitinated proteins. With the exception of the Met1 inter‐ubiquitin linkage, ubiquitination occurs via the ε‐amino group of a lysine residue on a target protein which is linked to the C‐terminus of the incoming ubiquitin [‐leucine (Leu)‐arginine (Arg)‐Gly‐Gly]. Ubiquitinated proteins therefore possess unique footprints following tryptic digest, which is commonly employed to process proteins for highly sensitive proteomic analysis by mass spectrometry, in the form of specific peptides containing the di‐glycyl lysine (Gly‐Gly‐ε‐Lys) motif with each peptide identifying a ubiquitination site 156. This motif is a general feature throughout all types of ubiquitin modification; in fact, all types of Ub‐like modifications as well which has to be kept in mind when, for example, sumoylation or neddylation also need to be considered. Thus, anti‐diglycyl lysine (in short, anti‐GGK or anti‐K‐ε‐GG) antibodies can be used to specifically concentrate Ub‐ and Ub‐like‐modified peptides. For example, pools of ubiquitinated peptides can be pulled out with the antibody after tryptic digestion, and systemic abundance of each ubiquitin linkage type is quantitatively measurable by mass spectrometry 157. Thereby, fold changes in the abundance between differently treated samples can be determined. Isotope labeling methods, such as stable isotope labeling with amino acids in cell culture (SILAC) 158, allow this comparative analysis. With isotope‐labelled reference peptides absolute abundance can also be determined which is known as absolute quantification method 159.

Multiple ubiquitin sensors have been constructed, both for polyubiquitins in general and for linkage‐specific polyubiquitins. Initially a sensor termed TUBE, which stands for tandem repeated ubiquitin entities, was developed by tandemly combining the sequence of UBA of ubiquilin 1 and human HR23A (UBA1) 160. Based on TUBE, linkage‐specific sensors were constructed by replacing its ubiquitin binding sites with Ub‐linkage‐specific domains such as, e.g. the ZFN of TAB2 and the UBAN motif of NEMO which detect and enrich Lys63‐linked and Met1‐linked ubiquitination, respectively 40, 67, 117. Employing a probe with the UBAN motif of NEMO, combined with SILAC‐based mass spectrometry, RIPK2 was identified as a LUBAC target in NOD2 signaling 67. Since the inactive catalytic domain of OTULIN has a 20‐fold or higher affinity (*K*_*d*_ = 100–200 nM) toward Met1‐linked di‐Ub than NEMO's UBAN motif (*K*_*d*_ = 3.7 μM) 100, this might turn out to be an even more sensitive probe for linear ubiquitin and, thus, LUBAC substrates than the UBAN motif of NEMO.

Even after the affinity purification, there is always an issue with non‐specificity. To address this and to confirm pulled‐down linkage types of certain ubiquitinated proteins, linkage‐specific and broad‐spectrum DUBs can help to answer the question of specificity when employed in parallel and together on affinity‐purified targets. Recently, Hospenthal *et al*. 161 proposed a sophisticated DUB toolbox system, termed UbiCRest to specifically decompose tangled ubiquitin linkages. For linear ubiquitination analysis, OTULIN is a potent tool, as reduction in ubiquitination on a particular protein following treatment by OTULIN corresponds to the amount of Met1‐linked ubiquitin present on this protein and, thereby, confirms identification of such a protein as a target of linear ubiquitination. This can be further combined with other DUBs, such as AMSH, as performed in the before‐mentioned study by Emmerich *et al*. 40, who thereby identified the presence of heterotypic Met1‐ and Lys63‐ linkage‐containing chains on components of the TLR/IL‐1R signaling pathways.

## Concluding remarks

Ubiquitination plays a crucial role in regulating innate and adaptive immunity. Past studies focused on Lys48‐ and Lys63‐linked ubiquitin chains in these signaling and revealed the importance of ubiquitination. Now, the regulation of signaling by ubiquitination emerges to be more complex as linear ubiquitination, as well as Lys11‐linked ubiquitination, have recently entered the picture. Linear ubiquitination is now widely recognized as indispensable for the orchestration of immune signaling, albeit always in cooperation with other types of ubiquitination.

Linear ubiquitin in signaling is created by LUBAC and removed by linear‐ubiquitin‐specific DUBs. Currently, it is thought that this is reversed by OTULIN, and perhaps by CYLD. Linear ubiquitin chains present in signaling complexes are sensed by specific interacting proteins via their Met1‐di‐Ub‐binding domains. We are only beginning to decipher the precise roles of particular linear ubiquitination events on specific target proteins with regard to recruitment and possibly also activation of the proteins recruited to them. It will be exciting to fill the gaps in our knowledge of these processes to gain a better understanding of the regulation of innate and adaptive immunity.
